# Stammering

**Published:** 1901-08-17

**Authors:** 


### STAMMERING.

WHATEVER may have been the original cause of stammering in any given case, long before treatment is sought it has become a trick or habit which is strongly engrained in the nervous system, and it is this trick that has to be conquered. Dr. Hamilton Graham Langwill [1] says that the first thing in treatment is to explain to the patient the double character of the mechanism of speech, involving as it does the arts of vocalisation and articulation. The stammerer should then be made to understand that his defect consists essentially in want of the proper harmony between the workings of these two mechanisms. In a very large majority of cases the main error lies in the fact that so much attention is directed by the patient to the articulation of words that their vocalisation is neglected, and it is therefore to the treatment of this the commonest type of stammering that Dr. Langwill directs attention. Should the stammerer have a musical ear the treatment of his condition will be considerably facilitated; but even the most unmusical person will usually recognise that when "singing," however unmelodiously, the words come with distinctly less difficulty than when speaking in his ordinary conversational tone. In singing he has, in fact, turned his attention to vocalisation instead of to articulation alone. If the patient is destitute of any ear for music he may adopt the method of intoning, as in the church services, not of course for permanent use, but in order that he may have firmly stamped on his mind the all-important part played by the cultivation of the vocal element in the cure of stammering. In the same way much help may in some cases be obtained by imitating the style and more especially the tones of someone with whose voice the speaker is familiar, and when reading aloud by adopting a "voice" different from the natural one. With the attention thus fixed upon the sound of the sentences the articulation of them becomes much less of a difficulty. The patient having been made thoroughly to understand the conditions involved in the production of speech, and having been fully impressed with the necessity of giving full play to the vocal as contrasted with the articulatory mechanism, he must then be taught carefully the factors involved in the production of the various vowel and consonantal sounds, as given by Professor Wyllie.[2] The patient should be encouraged to speak with a full voice, and in specially difficult cases to practise speaking in a fixed key or intoning. Then having thoroughly mastered what may be called the physiological alphabet—that is, the method of producing the various sounds represented by the alphabet—he should be made to learn certain sentences which have been arranged by Professor Wyllie so as to bring out the different characters of tone used in speech. In using these sentences he should be told to notice which sounds give him the greatest difficulty, and he should not only practise repeatedly the sentences containing these sounds, but should be encouraged to draw up further illustrative ones containing the difficult letters. The sentences to which he refers which are quoted from Professor Wyllie are very ingeniously arranged to contain the various initials which commonly give rise to difficulty. He should read aloud daily for ten or fifteen minutes, always commencing with the "stammerer sentences" and any exercises which have been arranged for the specially difficult consonants, and then should proceed first to poetry and then to prose, and all the time he should speak and read "with voice, making music with the voice, and listening to it as he speaks." The article we quote from is well worth reading by those interested in the matter; but the key to the matter seems to be that the stammerer should make vocalisation his first care and then intelligently build up the articulation required.

[1] Brit. Med. Jour., July 20. [2] Disorders of Speech, by Professor Wyllie.

